# Computational and modeling approaches for US threshold genetic evaluations of calving ease

**DOI:** 10.3168/jdsc.2025-0853

**Published:** 2025-11-13

**Authors:** J.M. Tabet, M. Bermann, D. Lourenco, A. Legarra

**Affiliations:** 1 Department of Animal and Dairy Science, University of Georgia, Athens, GA 30602; 2 Council on Dairy Cattle Breeding, Bowie, MD 20716

## Abstract

The US dairy calving ease (CE) genetic evaluation is based on a threshold sire-maternal grandsire (SMGS) model and includes 2 genetic components: one reflecting the sire's direct genetic effect on calving, and the other capturing the maternal influence, modeled through either the dam or the maternal grandsire. This study compared 2 CE evaluation models—SMGS and sire-maternal (SMAT)—using different solving algorithms: Newton-Raphson (NR) and expectation maximization (EM). The analysis used over 24 million CE records provided by the Council on Dairy Cattle Breeding. Correlations of GEBV for phenotyped sires and maternal grandsires were highly consistent across algorithms, exceeding 0.99 within models. The NR algorithm was the most computationally efficient solver, requiring fewer iterations and less computing time than EM. Both SMGS and SMAT models are suitable for routine genetic evaluation of CE in US dairy cattle, with NR and EM offering reliable and efficient solutions for single-trait analysis.

## Highlights

• The US calving ease genetic evaluation is a sire-maternal grandsire threshold evaluation.
• Sire-maternal grandsire and sire-maternal models produce highly correlated genomic estimated breeding values.
• Newton-Raphson and expectation-maximization give similar predictions within the same model.

## Graphical Abstract

**Figure:**
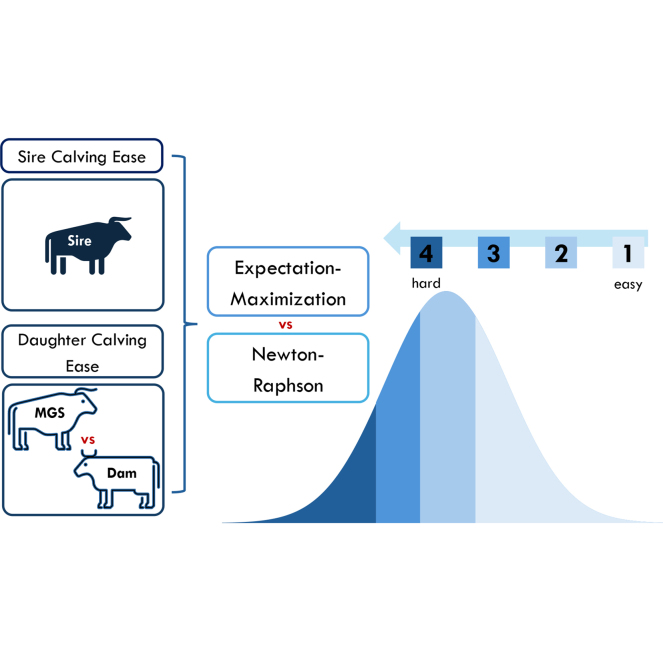
**Summary:** We proposed 2 models for the US calving ease genetic evaluation, each tested with 2 different threshold model solvers. Both models produced consistent correlations, and the solvers yielded comparable results, with the Newton-Raphson-based solver demonstrating faster computational performance. MGS = maternal grandsire.

The US national dairy genetic evaluations have included calving ease (**CE**) since 2002. It is directly scored by herd owners on a scale from 1 (easy calving) to 4 (extreme difficulty). After initial evaluations based on a sire threshold model (Berger, 1994), genetic evaluations for CE adopted the sire-maternal grandsire (**SMGS**) model to improve prediction accuracy by accounting for maternal effects, allowing breeding companies to incorporate these maternal effects into their selection programs (Van Tassell et al., 2003). The CE evaluation includes 2 genetic traits for a single phenotype. Sire calving ease (**SCE**) reflects the genetic influence of the sire on calving difficulty of the offspring calf, representing the sire's genetic effect of the model, whereas daughter calving ease (**DCE**) measures a cow's ability to calve easily. Breeders choose bulls with improved SCE semen straws (e.g., to inseminate heifers), whereas an easy calving herd is composed of daughters from bulls with good DCE. The DCE can be modeled either through the maternal grandsire (**MGS**) or the dam (Ramirez-Valverde et al., 2001). In the United States, both traits' PTA are reported to producers as the percentage of difficult births attributed to their respective genetic contribution.

The threshold model, introduced by Wright (1934), links categorical traits to an underlying normally distributed variable (liability), where observed categories are determined by thresholds on the liability scale. Hoeschele et al. (1995) developed an algorithm for genetic evaluation of a categorical trait together with several continuous traits, using a Newton-Raphson (**NR**) algorithm. An alternative solving approach proposed by Wang et al. (1997) uses the expectation maximization (**EM**) algorithm. Both algorithms are implemented in the program BLUP90IOD3, part of the BLUPF90 software suite (Misztal et al., 2014). The NR algorithm uses a set of mixed model equations (**MME**) that consider the normal scores, which are a function of observed categories and current solutions. These MME change at each round. The EM algorithm, at each round of EM, solves the linear MME and then uses the solutions to “impute” (in an EM manner) unobserved liabilities, which serve as pseudophenotypes in the next round of solving. The MME do not change at any round. Advantages of the EM method are that programming is greatly simplified and that it can be readily extended to multiple categorical traits; the inconvenience is that thresholds are assumed to be known. Moreover, EM requires more rounds to convergence than NR, and therefore, the convergence times might be inadequate. The objectives of this research were, first, to compare the computational performance and evaluation outcome of 2 threshold model solvers, NR versus EM, and second, to compare 2 model structures: SMGS versus sire-maternal (**SMAT**) for CE. We used records of the US national dairy genetic evaluation system. We assumed that models were correct and did not try to verify its fit by forward validation or similar methods.

Data for this study were provided by the Council on Dairy Cattle Breeding (**CDCB**; Bowie, MD), which is a subset of the official US dairy genetic evaluation for CE. The current CE evaluation is a SMGS model for joint genetic evaluation of Brown Swiss (**BS**) and Holstein (**HO**) bulls, incorporating records from purebred BS and HO cows. The initial dataset included 24,188,312 CE records (i.e., calving records rather than unique calf IDs; each record contains the sire and dam or MGS linked to the calving event; calf ID themselves are not recorded), with 82.6% of records classified as category 1 (easy calving), 9.1% as category 2 (long labor without assistance), 5.7% as category 3 (minimal assistance), and 2.6% in category 4 (extreme difficulty).

First, we ran a SMGS model, defined as

[1] $$\begin{array}{c} \lambda =X\beta +Z_{hys}hys+Z_{s}s+Z_{mgs}mgs+e \end{array},$$

where **λ** is the vector of CE liabilities, **β** is the vector of fixed effects comprised of year-season, breed-parity, and breed-sex, **hys** is the vector of random herd-year-season effects with variance $\left(\sigma_{hys}^{2}\right)$ equal to 0.216, **s** is the vector of random sire (of calf) genetic effect for the trait SCE with variance $\sigma_{s}^{2},$ **mgs** is the vector of random MGS (of calf) genetic effect for the trait DCE with variance $\sigma_{mgs}^{2},$ and **e** is the vector of residuals with variance $\left(\sigma_{e}^{2}\right)$ equal to 1.00. **X**, **Z_hys_**, **Z_s_**, **Z_mgs_** are incidence matrices relating the liabilities to **β**, **hys**, **s**, and **mgs**, respectively. The covariance structure for genetic effects is

[2] $$\begin{array}{c} \left(\begin{array}{c} s\\ \mathrm{mg}s \end{array}\right)\&sim;N\left(0,\left(\begin{array}{cc} \sigma_{s}^{2} & \sigma_{s,mgs}\\ \sigma_{mgs,s} & \sigma_{mgs}^{2} \end{array}\right)⊗H\right)=\;\left(0,\left(\begin{array}{cc} 0.0223 & 0.0113\\ symm. & 0.0224 \end{array}\right)⊗H\right) \end{array},$$

where $\sigma_{s,mgs}$ is the covariance between **s** and **mgs**, and **H** is the matrix combining pedigree and genomic relationships (Legarra et al., 2009; Aguilar et al., 2010; Christensen and Lund, 2010). The variance component estimates were provided by CDCB. Thresholds under the SMGS model were assumed to be known and obtained from CDCB estimates: $\begin{array}{ccc} {}[-1.14\; & -0.63\; & \;0.00] \end{array}.$ Note that not all thresholds are estimable. In this model, the restriction applied is that the last threshold is set to 0, whereas a more common approach is to fix the first threshold to 0. Both restrictions are equivalent and lead to the same predictions.

The pedigree used was an animal-based pedigree, formed by animal (sire), father (sire of sire), and mother (dam of sire). The model is therefore similar to a sire model (Mrode and Pocrnic, 2023), in that it assigns records to the sires of calves and the MGS of dams. However, it does not rely on an animal-sire-maternal grandsire pedigree structure, and so the specific rules to construct the SMGS pedigree relationship matrix (**A**) do not apply. The pedigree was constituted by flagging all sires (n = 68,672) and MGS (n = 159,268) in the data, then tracing back all their ancestors (males and females). The size of the pedigree was 702,367; of this, a total of 46,834 animals were genotyped (HO and BS), all imputed to a standardized set of 69,200 selected SNP, following the CDCB procedures as described at https://uscdcb.com/genomic-evaluations/.

An alternative sire-maternal (SMAT) model was proposed, in which the dam of the calf replaces the maternal grandsire to represent the DCE trait:

[3] $$\begin{array}{c} \lambda =X\beta +Z_{hys}hys+Z_{s}s+Z_{d}d+e, \end{array}$$

where **d** is the vector of random dam (of calf) genetic effect with $\sigma_{d}^{2},$ and **Z_d_** is the corresponding incidence matrix. The covariance structure for the genetic effects was reparametrized as $\sigma_{d}^{2}=4\sigma_{mgs}^{2}$ to reflect the dam effect:

[4] $$\begin{array}{c} \left(\begin{array}{c} s\\ d \end{array}\right)\&sim;N\left(0,\left(\begin{array}{cc} \sigma_{s}^{2} & \sigma_{s,d}\\ \sigma_{d,s} & \sigma_{d}^{2} \end{array}\right)⊗H\right)=\;\left(0,\left(\begin{array}{cc} 0.0239 & 0.0242\\ symm. & 0.0961 \end{array}\right)⊗H\right). \end{array}$$

Thresholds and $\;\sigma_{hys}^{2}$ were also rescaled under the SMAT model: $\begin{array}{ccc} {}[-1.22 & -0.67 & 0.00] \end{array}$ and $\sigma_{hys}^{2}=0.232.$

The pedigree was constituted by flagging all sires (of calves) and dams (of calves) in the data (n = 12,195,135), then tracing back all their ancestors (males or females). The final size of the pedigree was 19,684,850, of which 887,679 were genotyped using the same standardized set.

Genomic estimated breeding values were obtained using either EM or NR, as described earlier. Both have an outer loop that stops at convergence of EBV solutions. The EM algorithm requires thresholds to be known, and these had been previously estimated by CDCB using NR. Both EM- and NR-based solvers use variance components in the liability scale that were also known from previous studies.

We conducted a single-trait joint evaluation of BS and HO populations under single-step genomic BLUP (Aguilar et al., 2010) with Metafounders (Legarra et al., 2015). In addition to evaluating the 2 estimation methods (NR vs. EM), we also compared the performance of the SMGS and SMAT models. Several metrics were calculated. First, we calculated the Pearson correlation between GEBV of phenotyped sires and MGS for both SCE and DCE, across models and solvers. For DCE specifically, we compared the solutions of phenotyped MGS between the SMGS and SMAT models. We also recorded the number of inner loop and outer loop iterations needed to reach convergence. In addition, we reported the elapsed wall-clock time (in hours) for each analysis. These results allowed us to evaluate the impact of solver type, model changes, and convergence criteria on prediction. In principle, the SMAT model is expected to be more accurate than the SMGS model because it incorporates more information. However, this also means that a direct comparison of computational performance between the 2 models cannot be made. Additionally, the NR and EM solvers should yield the same solutions, and for practical purposes, one needs a model and solver as accurate and computationally fast as possible.

Pearson correlations of GEBV for phenotyped sires and MGS in SCE and DCE are presented in Table 1. Under the SMGS model, correlations among NR and EM were 0.99 for both SCE (upper diagonal quadrant) and DCE (lower diagonal quadrant), so they yielded the same solutions, as expected. A similar pattern was observed with the SMAT model. When comparing across models (SMGS vs. SMAT), correlations were 0.98 for SCE and 0.96 for DCE, among all solvers. These differences were expected because we changed the evaluation model (SMGS vs. SMAT) for sire and MGS evaluations.

**Table 1:** Pearson correlations among GEBV; upper diagonal quadrant shows correlations of sire calving ease (SCE) for phenotyped sires, and lower diagonal quadrant shows correlations of daughter calving ease (DCE) for phenotyped maternal grandsires

Model	NR-SMGS	EM-SMGS	NR-SMAT	EM-SMAT
NR-SMGS	**1.00**	0.99	0.98	0.98
EM-SMGS	0.99	**1.00**	0.98	0.98
NR-SMAT	0.96	0.96	**1.00**	0.99
EM-SMAT	0.96	0.96	0.99	**1.00**

^1^NR-SMGS = Newton-Raphson (NR) with Sire-Maternal Grandsire model; EM-SMGS = Expectation Maximization Sire-Maternal Grandsire model; NR-SMAT = NR with sire-maternal model; EM- SMAT = EM with Sire- Maternal model.

Figure 1 presents the distributions of GEBV for SCE under the SMGS and SMAT models, using the NR and EM algorithms. The distributions were similar across algorithms, suggesting that the different solvers produced comparable solutions. A similar trend was observed for DCE under each model (not shown).

**Figure 1:**
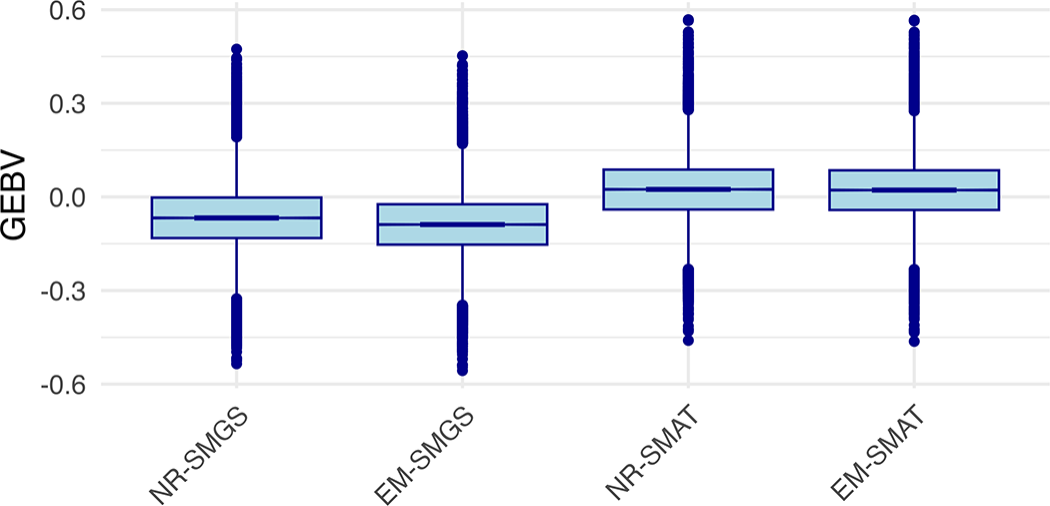
Distribution of GEBV for phenotyped sires and maternal grandsires for sire calving ease under sire–MGS (SMGS) and sire–maternal (SMAT) models solved by Newton–Raphson (NR) or expectation–maximization (EM). Boxplots: center line = median; box = Q1–Q3; whiskers = 1.5 × interquartile range; points = outliers.

Table 2 summarizes the computational performance of each algorithm under the 2 evaluation models. For the SMGS model, NR proved to be much faster than EM, requiring around 100 fewer outer iterations and about 700 fewer inner iterations to reach convergence. This difference is expected, given that NR is generally faster than EM (e.g., Wang et al., 1997). Similar trends were observed under the SMAT model, with NR again requiring fewer inner (∼2,000) and outer (∼100) iterations than EM.

**Table 2:** Number of inner iterations (inner) and outer iterations to convergence (outer), and total wall-clock time (in hours) for each combination of method (solver) and model used in the calving ease evaluations

Model^2^	Method^1^					
	NR			EM		
	Inner	Outer	Time	Inner	Outer	Time
SMGS	1,064	8	1.62	1,989	117	4.63
SMAT	2,966	9	9.27	4,562	110	20

1 NR = Newton-Raphson (NR); EM = expectation maximization.

2 SMGS = sire-maternal grandsire model; SMAT = sire-maternal model.

In terms of computing time, SMGS-EM completed in 4.63 h, compared with NR, which required 1.62 h to reach convergence. Under SMAT, this difference was more apparent, as NR took 9.27 h to finish, compared with 20 h for EM; this difference is attributed to the larger amount of data used in the SMAT model. From a practical perspective, a total computing time of 1 d or less is generally acceptable. All SMGS models ran in less than 1 d, as did the SMAT model using NR.

Incorporating a maternal effect in the model improves accuracy by accounting for the dam's maternal ability (Van Tassell et al., 2003). In our study, representing the maternal effect using either the MGS (SMGS) or the dam (SMAT) led to similar genetic evaluations for the phenotyped sires and MGS. Ramirez-Valverde et al. (2001) reported similar results when comparing a sire model with MGS effect to an animal model with maternal effect under threshold models. Generally, a model including dam instead of MGS is more accurate because the relationship across animals includes relationships via mothers of dams, and the MGS is still included in the model as the father of the dams. Our analysis did not use a full animal model, because the “direct” genetic effect in this context refers to the calf's sire rather than the calf itself. This reflects the industry's focus on attributing CE scores directly to the service sire, which is more practical for management. This approach is to improve prediction accuracy for service sires based on field-recorded CE data. In turn, predictions for the dam or MGS help select cows that “calve easily.”

In terms of computational efficiency, the SMGS model required solving fewer equations, as it included only animals with CE records and their ancestors, resulting in shorter runtimes compared with the SMAT model, which evaluated a much larger number of animals due to the greater number of dams relative to MGS.

Our results suggest that both NR and EM algorithms produce similar predictions across models. The fastest option is NR, but as of now, there are no algorithms to extend it to multiple categorical traits.

We conclude that both SMGS and SMAT models can be used for the US dairy genetic evaluation of CE, as no differences were observed in SCE or DCE for phenotyped sires and MGS. This study also evaluated the computational efficiency of 2 solvers: NR and EM. Both algorithms produced equivalent results. For practical implementation, the most efficient and reliable approach for single-trait evaluation of categorical data is the NR solver.

## References

[bib1] Aguilar I., Misztal I., Johnson D.L., Legarra A., Tsuruta S., Lawlor T.J. (2010). Hot topic: A unified approach to utilize phenotypic, full pedigree, and genomic information for genetic evaluation of Holstein final score. J. Dairy Sci..

[bib2] Berger P.J. (1994). Genetic prediction for calving ease in the United States: Data, models, and use by the dairy industry. J. Dairy Sci..

[bib3] Christensen O.F., Lund M.S. (2010). Genomic prediction when some animals are not genotyped. Genet. Sel. Evol..

[bib4] Hoeschele I., Tier B., Graser H.-U. (1995). Multiple-trait genetic evaluation for one polychotomous trait and several continuous traits with missing data and unequal models. J. Anim. Sci..

[bib5] Legarra A., Aguilar I., Misztal I. (2009). A relationship matrix including full pedigree and genomic information. J. Dairy Sci..

[bib6] Legarra A., Christensen O.F., Vitezica Z.G., Aguilar I., Misztal I. (2015). Ancestral Relationships using metafounders: Finite ancestral populations and across population relationships. Genetics.

[bib7] Misztal I., Tsuruta S., Lourenco D., Masuda Y., Aguilar I., Legarra A., Vitezica Z.G. (2014). Manual for BLUPf90 family of programs. https://nce.ads.uga.edu/html/projects/programs/docs/blupf90_all8.pdf.

[bib8] Mrode R., Pocrnic I. (2023).

[bib9] Ramirez-Valverde R., Misztal I., Bertrand J.K. (2001). Comparison of threshold vs linear and animal vs sire models for predicting direct and maternal genetic effects on calving difficulty in beef cattle. J. Anim. Sci..

[bib10] Van Tassell C.P., Wiggans G.R., Misztal I. (2003). Implementation of a sire-maternal grandsire model for evaluation of calving ease in the United States. J. Dairy Sci..

[bib11] Wang C.S., Quaas R.L., Pollak E.J. (1997). Bayesian analysis of calving ease scores and birth weights. Genet. Sel. Evol..

[bib12] Wright S. (1934). An analysis of variability in number of digits in an inbred strain of guinea pigs. Genetics.

